# The biologically functional identification of a novel TIM3-binding peptide P26 in vitro and in vivo

**DOI:** 10.1007/s00280-020-04167-0

**Published:** 2020-10-21

**Authors:** Tangwu Zhong, Chuanke Zhao, Shuntao Wang, Deshuang Tao, Shuxia Ma, Chengchao Shou

**Affiliations:** 1 School of Basic Medicine, Jiamusi University, 258 Xuefu Street, Jiamusi, 154007 Heilongjiang Province China; 2 Key Laboratory of Carcinogenesis and Translational Research (Ministry of Education/Beijing), Department of Biochemistry and Molecular Biology, Peking University Cancer Hospital and Institute, 52 Fucheng Road, Beijing, 100142 China; 3 Jiamusi Central Hospital, Jiamusi, 154002 Heilongjiang Province China

**Keywords:** P26, TIM3, Phage display peptide library, Biological activity, Anti-tumor

## Abstract

**Purpose:**

Recent studies have shown that TIM3 plays an important role in T-cell failure, which is closely related to the resistance to anti-programmed cell death protein 1 (PD-1) treatment. However, there have been no reports on the application of peptide blockers to TIM3. In this study, we endeavored to identify the in vitro and in vivo anti-tumor activities of a TIM3-targeting peptide screened from the phage peptide library.

**Methods:**

Phage display peptide library technology, surface plasmon resonance, flow cytometry, and mixed lymphocyte reaction were utilized to screen and demonstrate the bioactivities of P26, a TIM3-targeting peptide. Meanwhile, tumor growth assay was performed to evaluate the anti-tumor effect of P26.

**Results:**

In terms of affinity, we demonstrated that P26 specifically binds to TIM3 at the cellular and molecular levels, which therefore blocks the interaction between TIM3 and Galectin-9 (Gal-9) and competes with Gal-9 to bind TIM3. Additionally, P26 significantly increases T-cell activity and elevates IFN-γ and IL-2 levels in a dose-dependent manner. Notably, P26 also counteracts Gal-9-mediated T-cell suppression. More importantly, P26 can inhibit growth of MC38-hPD-L1 tumor in mice.

**Conclusions:**

P26, as a novel TIM3-binding peptide, has the ideal bioactivity connecting to TIM3 and the potential prospect of application in immunotherapy as an alternative or adjuvant to existing agents.

## Introduction

Immunotherapy is increasingly recognized as one of the most promising treatments for cancer therapy, and there has been a focus on the development of treatments aiming at some immunological checkpoint molecules such as anti-cytotoxic T-Lymphocyte-Associated Protein-4 (CTLA-4) and PD-1 in melanoma, kidney cancer, Hodgkin's disease, and lung cancer [1]. However, tumors grow in the complex networks consisting of epithelial cells, blood vessels and lymphatic vessels, cytokines and chemokines, and infiltrating immune cells [2]. In some cancers including colorectal cancer, the blockade of CTLA-4 or PD-1 does not achieve an ideal therapeutic effect and incentivizes the efforts to locate other immune checkpoint inhibitors, such as TIM3. Recent studies have shown that TIM3 plays an important role in T-cell failure. The resistance to anti-PD-1 treatment is partly due to the up-regulation of TIM3 expression in T cells, and the use of anti-TIM3 antibodies in combination with anti-PD-1 inhibitors can override drug resistance [3, 4].

The *TIM* gene family contains eight members *(TIM1-8)* in mouse chromosome 11B1.1 and three members *(TIM1, TIM3,* and *TIM4)* in human chromosome 5q33.2 [5]. Human *TIM3*, also known as *HAVCR2*, is a type I membrane protein with 302 amino acids, of which 63% are identical to murine TIM**3** (281 amino acids) [6]. The main ligands of TIM3 are carcinoembryonic antigen cell adhesion molecule 1 (CEACAM-1), phosphatidylserine (PS), high-mobility group protein B1 (HMGB1), and Galectin-9 (Gal-9) [3], among which Gal-9 was the first being identified and its interplay with TIM3 triggers an immunosuppressive outcome through the activation of JUN N-terminal kinase (JNK) and AKT signaling pathways [7]. Some of the latest findings have shown that TIM3 acts as a biomarker that represses the spontaneous immune reactions in the high grade serous ovarian cancers (HGSC) [8], and up-regulation of TIM3 and regulatory T-cell infiltration could mediate the resistance to radiation therapy and PD-L1 blockade [9].

Phage display technology, as a selection-based system, is a well-used biological method and has been successfully applied to various fields [10]. We previously identified peptides blocking VEGF–VEGFR-1 interaction using this technique [11–13]. In the study, we focused on screening the peptides specifically targeting TIM3. Here, four rounds of subtractive screening were performed using the 293T cells stably expressing human TIM3 and the 12-mer phage display peptide library to identify the TIM3-binding peptides. A novel high-affinity TIM3-targeting peptide with excellent biological activities, P26, was identified. P26 can compete with Gal-9 for binding to TIM3 and diminish the biological functions of TIM3/Gal-9. P26 also exhibits the anti-tumor function in vivo. These results suggest that P26 may have potential applications as an immunological checkpoint inhibitor or supplementation to other immune checkpoint inhibitors.

## Materials and methods

### Reagents

The Ph.D.-12 phage display peptide library kit was purchased from New England Biolabs. Histopaque-1077 (10771) was from Sigma-Aldrich. CD4^+^ T-cell isolation kit (130-096-533) and Mo-DC Generation Toolbox I (130-093-568) were obtained from Miltenyi Biotech. The Human IFN-gamma DuoSet ELISA (DY285B) and Human IL-2 DuoSet ELISA (DY202) were from R&D Systems. The TMB substrate single solution (D0022) was purchased from Yacoo. Opdivo (NDC 0003-3772-11) was obtained from Bristol-Myers Squibb. Gal-9 fusion protein (CHI-HF-210GAL9) and TIM3 fusion protein (CHI-MF-210T3-C050) were obtained from Chimerigen. The anti-TIM3 monoclonal antibody C23 was generated by our laboratory. Anti-GAPDH (10494-1-AP) was purchased from Proteintech. HRP-anti-mouse (ab6789) was obtained from Abcam.

### Cell lines and cell culture

Human renal epithelial cell line 293T and 293T-TIM3 were obtained from KYinno and maintained in Dulbecco’s modified Eagle’s medium (DMEM) at 37 ℃, 5% CO_2_. The media were supplemented with 10% FCS (Gibco) plus Penicillin/Streptomycin. Mouse colon cancer cell line MC38-hPD-L1 was obtained from Biocytogen (Beijing, China) and cultured in RPMI-1640 plus 10% FBS (Gbico) and hygromycin B (100 μg/mL) at 37 ℃, 5% CO_2_. Mo-DC Differentiation Medium (130-094-812) and Mo-DC Maturation Medium (130-094-813) were purchased from Miltenyi Biotech.

### Western blot analysis

Cells were lysed in buffer containing 50 mM Tris–HCl pH 7.0, 150 mM NaCl, 2 mM EDTA, 2 mM dithiothreitol, 1% SDS, and 1 × protease inhibitor cocktail from Roche. The protein concentration was quantified by the BCA protein detection kit. Samples were separated by SDS-PAGE and electro-blotted to the nitrocellulose membranes, which were blocked with 5% non-fat milk in PBS for 1 h at room temperature, probed with the indicated primary antibodies at 4 ℃ overnight, and incubated with HRP-conjugated secondary antibody for 45 min at room temperature. Protein bands were visualized with the enhanced chemiluminescence system.

### Screening of TIM3-targeting peptides by phage display random peptide library

293T-TIM3 (target cells) and 293T (negative absorption cells) were used to screen the random phages display peptide libraries. Panning was performed by procedures suggested by Peptide Library Kit with some modifications. After amplification of the ultimate phage peptide library obtained by subtractive screening, one part of phage supernatant was subjected to next-generation sequencing (NGS, completed by Abace-Biology) to select frequently occurring peptide coding sequences. Another part was subjected to ELISA to select positive clones. Finally, preferred peptide sequences were selected based on the results of both NGS and ELISA. The candidate peptides and a control peptide (P0) were synthesized by Apeptide using standard solid-phase Fmoc chemistry. FITC conjugates were produced by DGpeptides.

### Examination of binding specificity and ligand competition binding assays

Surface plasmon resonance (SPR) assay. The affinity identification between P26 and TIM3 was performed by immobilizing the TIM3 protein (50 μg/mL) on the surface of the nanogold sensor chip after the chip was activated by EDC/NHS. P26/Gal-9 competition assay was performed by immobilizing Gal-9 protein (25 µg/mL) on the sensor chip. Each sample was injected at a flow rate of 20 μL/min for about 7 min. *KD*, *ka*, and k*d* were calculated by Trace Drawer Evaluation (version 2.0).

Flow cytometry. After blocking with 1% BSA-PBS for 30 min at room temperature and washing, 500 μL of cell suspension (2 × 10^6^ 293T-TIM3 or 293T cells) was incubated with FITC-conjugated P26 peptide and/or Gal-9. After incubation for 30 min at room temperature and washed twice with PBS, the cells were resuspended in 200 μL PBS for flow cytometry analysis.

### Isolation and purification of dendritic cells (DCs) and CD4^+^ T cells and mixed lymphocyte reaction (MLR)

The blood of donors was diluted (volume ratio 1:1) with physiological saline, and human peripheral blood mononuclear cells (PBMCs) were isolated with the human lymphocyte separation solution Histopaque-1077 by centrifugation at 400 g for 40 min at room temperature. The obtained PBMCs were washed for three times with PBS and subjected to magnetic bead sorting. CD4^+^ T cells were isolated from the remaining cells after isolation of DCs by the Mo-DC generation kit I and CD4^+^ T cell isolation kit. DCs were cultured in Mo-DC differentiation medium for 7 days and then replaced with Mo-DC mature medium for 3 days.

Mature DCs and newly isolated T cells (different donors) were mixed for MLR. About 1 × 10^4^ DCs and 1 × 10^5^ T cells per well were mixed and cultured in 200 µL RPMI-1640 medium plus 10% FBS. To evaluate the activity of P26 (gradient effect), competitive inhibition (P26 and Ligand), as well as synergistic effect (P26 and PD-1 inhibitor OPDIVO), indicated concentrations of peptide, OPDIVO, and Gal-9 were added to the corresponding wells to interact with the cells. The expression levels of IFN-γ or IL-2 secreted in the cell supernatant were measured on the fifth day by ELISA assay.

### ELISA assay for T-cell activity

IFN-γ and IL-2 in MLR cell supernatants were detected according to the ELISA kit procedure. Briefly, 100 μL of capture antibody solution (2 μg/mL) was coated in a 96-well plate overnight at room temperature. On the second day, the capture antibody solution was aspirated out, and the wells were washed three times with 300 μL of PBST containing 0.05% Tween 20. Afterwards, wells were blocked with 300 μL of 1% BSA-PBS for 1 h. After sealing, the wells were similarly washed and incubated with 100 μL of test samples or standards per well for 2 h. After that, each well was incubated with the detection antibody (125 ng/mL, 100 μL) for 2 h. After incubation with 100 μL of HRP-conjugated secondary antibody for 20 min, color was developed with TMB. The test wells were read at the wavelength of 450/570 nm using an ELISA plate reader (Bio-Rad).

### Tumor growth assay

C57BL/6 mice transgenic for B-hHAVCR2 (TIM3) (Biocytogen, Beijing, China) were used to evaluate the anti-tumor activity of P26 *in* *vivo*. After 1 week of adaptive feeding, 6-week-old female mice were inoculated with 0.1 mL of MC38-hPD-L1 cells (1 × 10^6^ cells) by subcutaneous injection on the right scapula followed by the measurements of tumor volume and body weight. When the average tumor volume reached about 80 mm^3^, mice were randomly divided into three groups and administrated with different drugs: (A) PBS (control), (B) ATE (PD-L1 inhibitor Atezolizumab, 10 mg/kg/4d, as the positive group), (C) P26 (5 mg/kg/d), and (D) P26 (5 mg/kg/d) plus ATE (10 mg/kg/4d). The weight and tumor volume of the mice were measured twice a week. When the body weight loss (% BWL) was above 20%, the drug was discontinued, and then, ethically acceptable execution to mice was adopted upon the failure of weight loss recovery in 72 h. The weight and volume of tumors were then analyzed.

### Statistics analysis

All the data were expressed as means ± SEM of duplicate or triplicate samples. Histograms and line charts were generated by GraphPad Prism 6.0. *T *tests or one-way ANOVA was used to determine the *p *values. Statistical significance was determined as a *p* value < 0.05.

## Results

### Screening of specific peptides binding to TIM3 from a random library

**Fig. 1:**
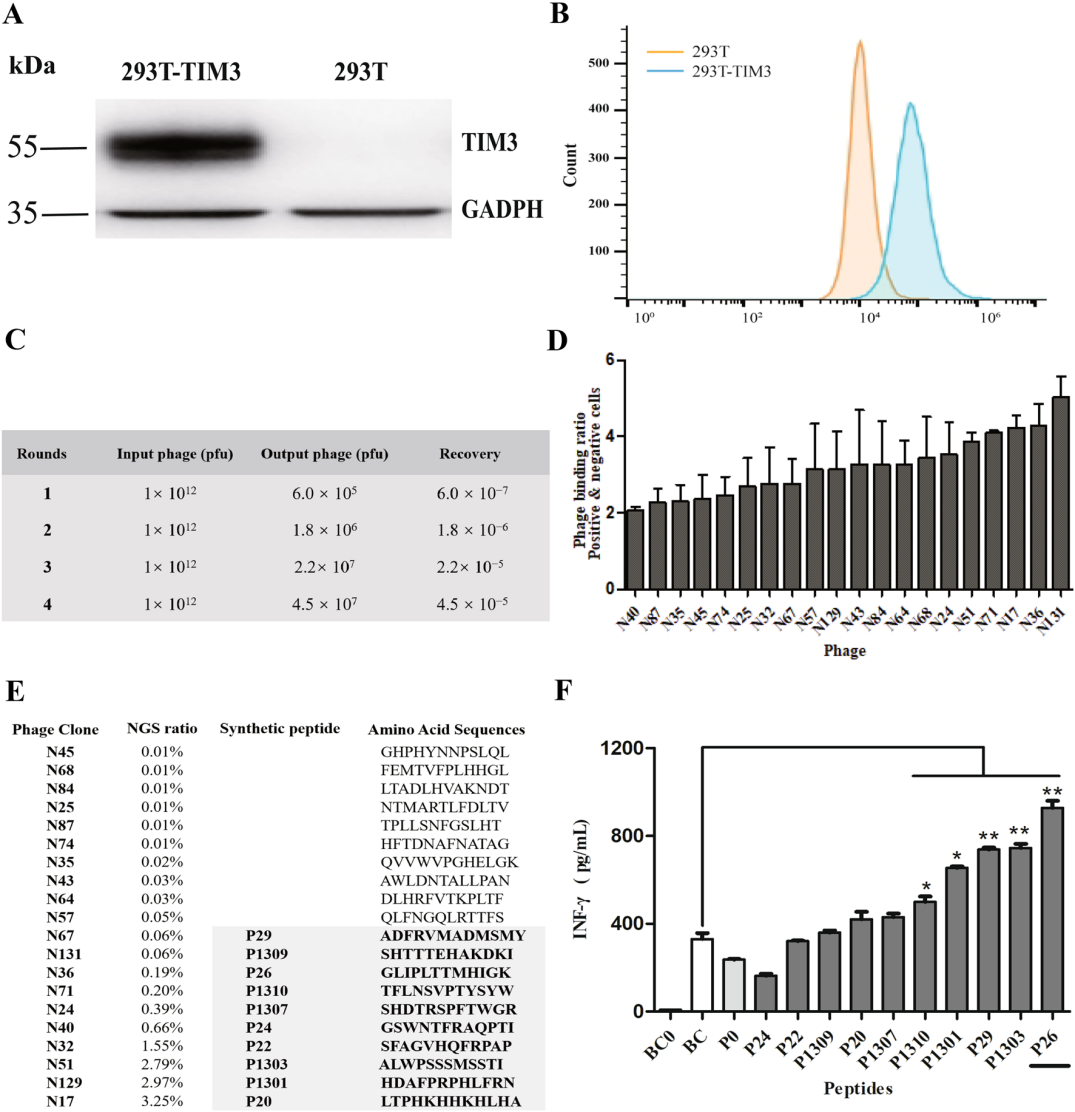
Screening and synthesis of TIM3-targeting peptides from a random Phage Display 12-mer-Peptide Library. **a** Stable expression of exogenous human TIM3 protein in 293T cells was detected by Western Blot. **b** Expression of TIM3 on the surface of 293T cells was detected by flow cytometry. **c** Recovery of positive clones of TIM3-binding phages from each round by subtractive bio-panning in the Phage Display 12-mer-Peptide Library. **d** Relative ratio of the OD490 value of the positive clone’s association with target cells (293T-TIM3) to the control cells (293T) was compared and analyzed by ELISA. **e** Amino acid sequences of 20 clones screened by NGS, and 10 peptides (boxed) were synthesized based on the results of ELISA and NGS. **f** Secretion of IFN-γ by peptides and cells incubation after co-culture. The BC0 group contained only T cells, the BC group contained only DCs and T cells, and the other groups were mixed cultures of peptides and DCs and T cells. Data were showed as mean ± SEM, *p < 0.05, **p < 0.01

293T-TIM3 and 293T cells were, respectively, used as the target cells and negative absorption cells to select a random phage display 12-mer-peptide library. The expression of *TIM3* in the 293T-TIM3 cell line was verified by Western blot analysis (Fig. 1a) and flow cytometry (Fig. 1b). The enriched phage clones showed a significant increase in relative yield during four rounds of step-by-step bio-panning (Fig. 1c). After the fourth round, one part of phage supernatant of the ultimate phage peptide library was subjected to NGS, and another part was subjected to ELISA. Twenty clones from the screen-amplified round 4 phage library were subjected to cell ELISA, and the relative ratios of the OD_490_ values of the positive clones (binding with 293T-TIM3 cells to binding with 293T cells) were compared and analyzed **(**Fig. 1d). Twenty frequently occurring peptide coding sequences were screened by NGS, and then, ten peptides were synthesized based on the results of NGS and phage ELISA for further study (Fig. 1e). The result showed that there was no homology or consensus motif in these clones. Next, we sought to test whether the peptides could activate CD4^+^ T cells. Peptides (100 μg/mL) were added to the co-culture of DCs and T cells, and ELSIA was proceeded to detect IFN-γ produced by DCs and T cells in cell supernatants during the co-culture. Peptide P26 with the strongest activity in inducing IFN-γ was selected for further analysis (Fig. 1f).

### Affinity of P26 to TIM3

**Fig. 2:**
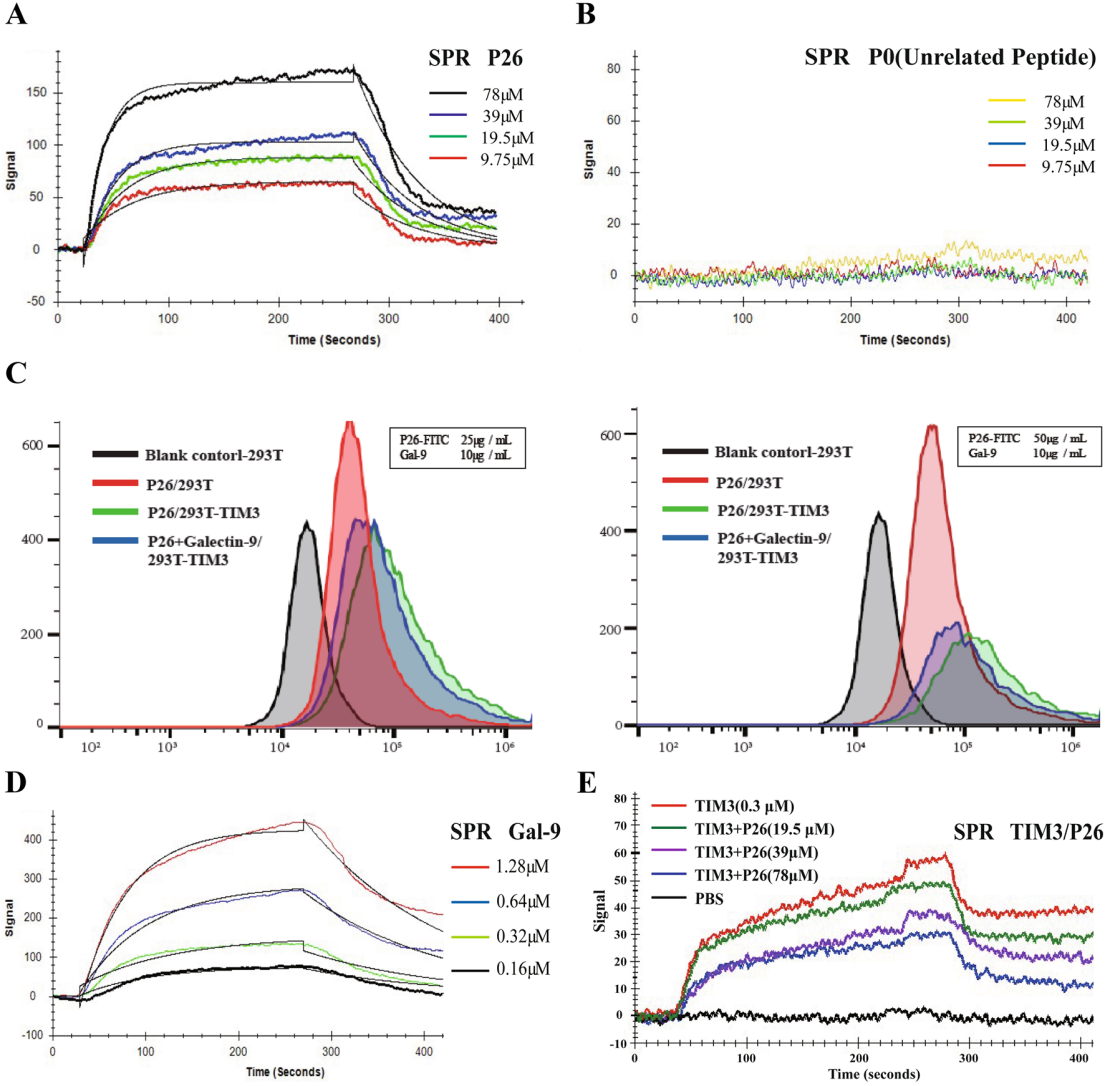
The binding affinity of peptide P26 to TIM3. **a** The KD value of P26 binding to TIM3 was examined by SPR (sensor chip of TIM3). **b** SPR-binding curves of negative control peptide P0 (sensor chip of TIM3). **c** The binding specificity of P26-FITC (25 µg/mL and 50 µg/mL) to293T-TIM3 cells and the capacities of FITC-P26 and FITC-Gal-9 (10 µg/mL) to compete for binding to 293T-TIM3 cells were examined by flow cytometry. **d** The KD value of Gal-9 binding to TIM3 was detected by SPR (sensor chip of TIM3). **e** Effects of indicated concentrations of P26 on Gal-9-TIM3 binding tested by SPR (sensor chip of Gal-9)

Affinity of P26 to TIM3 was examined by SPR and flow cytometry. In SPR assay, P26 dose-dependently associated with TIM3 and 3.44 × 10^–5^ M of affinity constant (*KD*), 4.94 × 10^–2^ Ms^−1^ of *ka*, and 1.70 × 10^–2^ s^−1^ of *kd* were obtained (Fig. 2a). No specific binding between P0 and TIM3 was found (Fig. 2b). In flow cytometry, P26 exhibited a higher binding curve to 293T-TIM3 cells than to 293T cell (Fig. 2c). Therefore, P26 can specifically recognize either free or adherent TIM3.

### P26 blocks the interaction between TIM3 and Gal-9

The competitive binding test between P26 and Gal-9 to TIM3 was also conducted by SPR and flow cytometry. The results of flow cytometry also indicated a competitive relationship between P26 and Gal-9′s bindings to 293T-TIM3 cells (Fig. 2c). In addition, the binding between Gal-9 and TIM3 was validated by SPR and the KD, ka, and kd values were 6.27 × 10^–7^ M, 1.07 × 10^–4^ Ms^−1^, and 6.71 × 10^–3^ s^−1^, respectively (Fig. 2d). However, P26 significantly decreased TIM3/Gal-9 interaction (Fig. 2e). Therefore, P26 can not only specifically bind to TIM3 but also compete with Gal-9, resulting in diminished TIM3/Gal-9 interaction.

### P26 activates T cells and reverses Gal-9-inhibited T-cell activation

**Fig. 3:**
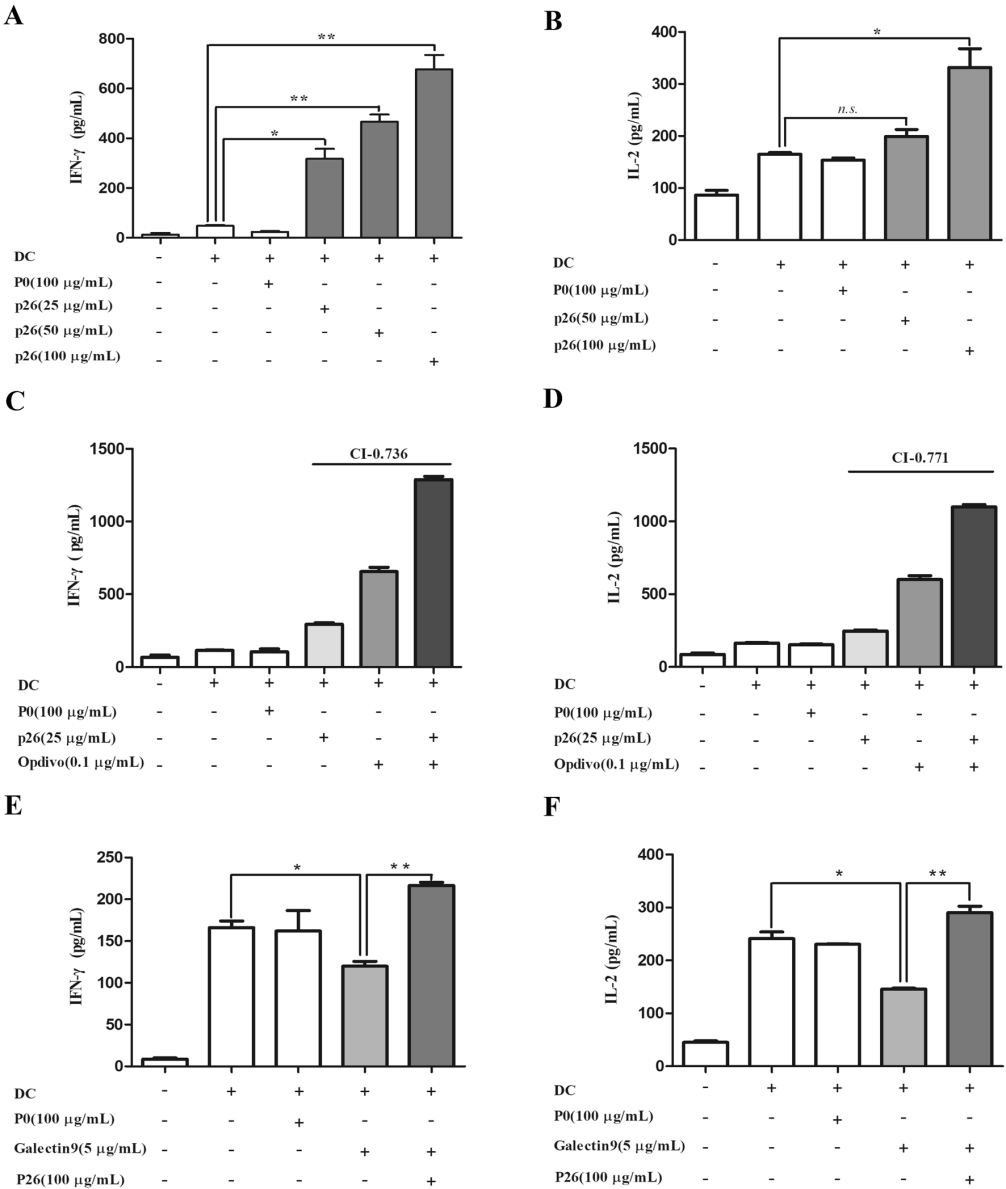
The secretion of IFN-γ and IL-2 after co-incubation of P26 and P26 with other drugs in MLR was detected by ELISA. **a** and **b** IFN-γ (**a**) and IL-2 (**b**) secretion levels after incubation of different concentrations of P26. **c** and **d** Secretion of IFN-γ (**c**) and IL-2 (**d**) after co-incubation of P26 (25 µg/mL) and PD-1 inhibitor OPDIVO (0.1 µg/mL). **e** and **f** Secretion levels of IFN-γ (**e**) and IL-2 (**f**) after co-incubation of P26 (100 µg/mL) and Gal-9 (5 µg/mL). Data were analyzed and presented as mean ± SEM, *p < 0.05, **p < 0.01. *n.s.* no significance

To evaluate the impact of P26 on T-cell activation and proliferation, MLR experiments were performed. The levels of IFN-γ and IL-2 in the supernatant were slightly increased after DC-cell presentation, but were markedly elevated by P26 in a dose-dependent fashion (Fig. 3a and b). Furthermore, P26 and the PD-1 inhibitor, OPDIVO, jointly stimulated T cells, and the combined indexes (CI) [14, 15] of P26 and OPDIVO were 0.736 for IFN-γ and 0.771 for IL-2, respectively (Fig. 3c and d**)**. In addition, we evaluated the competitive effect of P26 on TIM3/Gal-9-regulated T-cell activation. Gal-9 (5 µg/mL) reduced the levels of IL-2 and IFN-γ from DC-primed T cells. However, in the presence of P26, Gal-9-mediated inhibition on T-cell activation was abrogated (Fig. 3e and f). Therefore, P26 has the capacity to reverse Gal-9-inhibited T-cell activation.

### P26 inhibits tumor growth in vivo

**Fig. 4:**
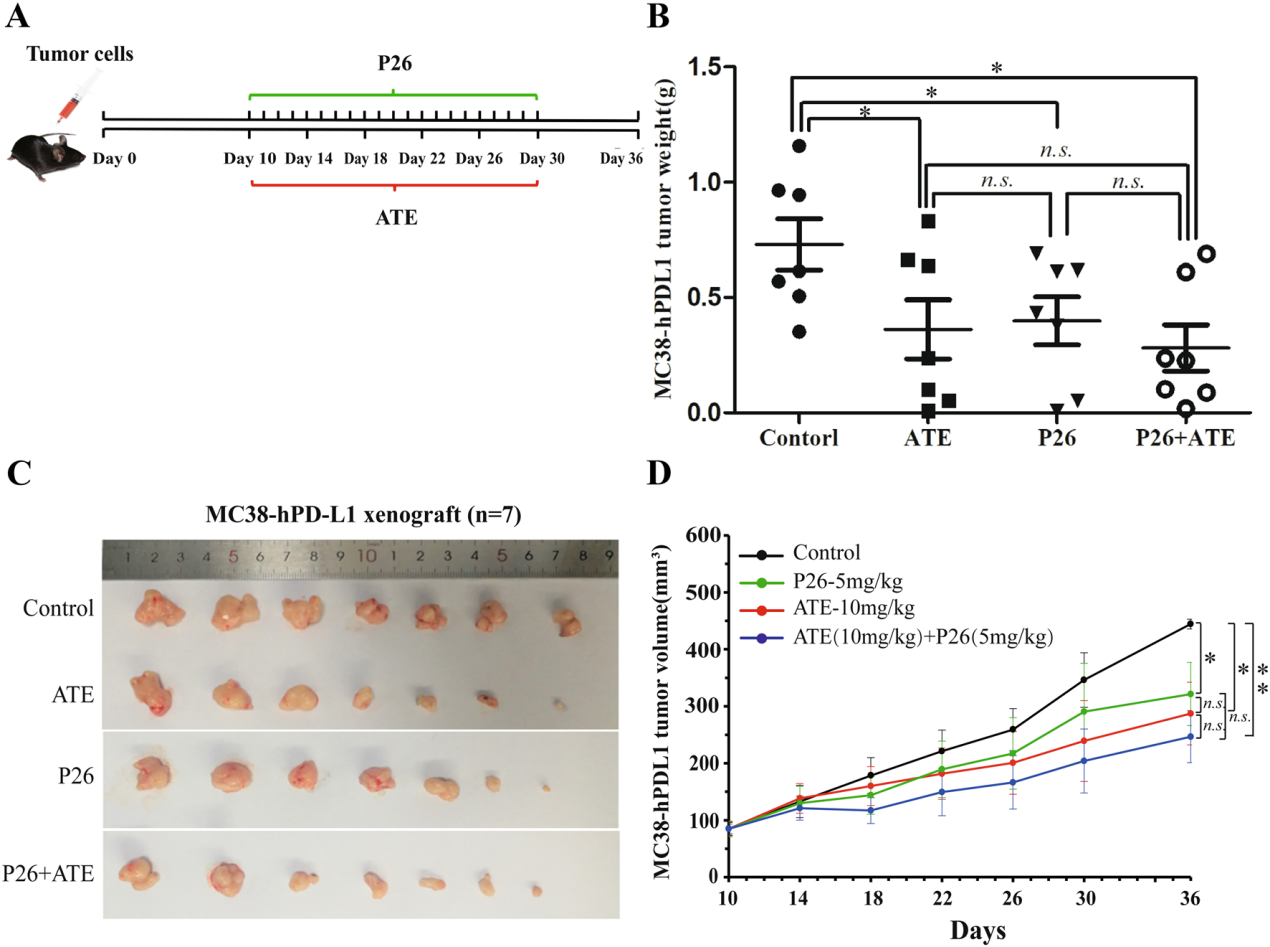
P26 inhibited tumor growth in mice. **a** Schema for subcutaneous implantation of MC38-hPD-L1 cells in mice on day 0, therapeutic injection of indicated drugs on day 10, and the mice were sacrificed on day 36. **b** and **c** Weight (**b**) and photos (**c**) of MC38-hPD-L1 tumor nodules on day 36. **d** Curves showing the changes of tumor volume. Data represent mean ± SEM, (*n* = 7). *p < 0.05, **p < 0.01. *n.s.* no significance

The MC38-hPD-L1 tumor-bearing B-hTIM-3 humanized mice model was used to evaluate whether P26 has an inhibitory effect on tumor growth in vivo. A dosing regimen was established (Fig. 4a), and the PD-L1 inhibitor ATE was used as a positive control. One week after withdrawal, tumor nodules were separated and then weighed for statistical analysis, the differences between the tumors in each group were compared, and tumor volume growth curves were shown. The results showed that, compared with the solvent control group, both P26 and ATE groups could significantly inhibit the growth of MC38-hPD-L1 tumor cells (Fig. 4b and c). In terms of tumor volume, the inhibition rates were 43.71% for ATE and 34.19% for P26 (Fig. 4d). The differences between P26 and ATE-inhibited tumor growth were statistically insignificant (Fig. 4b and d**)**. Likely due to the small sample size, the differences between co-treatment (P26 plus ATE) and single treatment (P26 or ATE) were insignificant, but combination of P26 and ATE did show a stronger inhibition on tumor weight (Fig. 4b) and volume (54.98%) (Fig. 4d). These results demonstrate that P26 has a considerably negative impact on tumor growth.

## Discussion

**Fig. 5:**
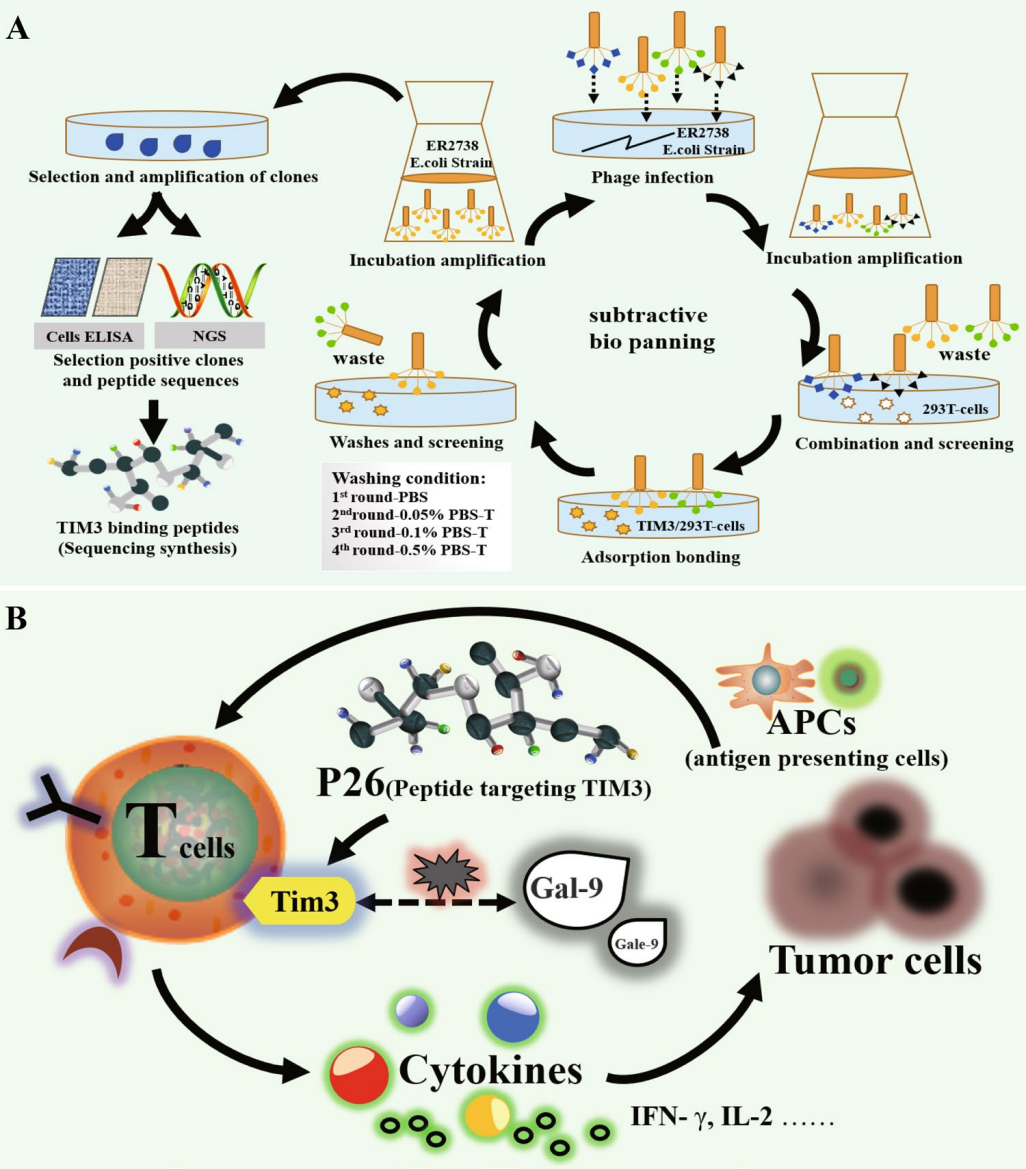
**a** Schematic diagram of the design and screening procedure for TIM3-binding peptides. **b** The mechanism by which the TIM3-binding peptide P26 activates T cells in a simulated organism environment

In this report, a panel of 12aa peptides targeting TIM3 were screened and identified by the phage peptide library display technology (screening procedures shown in Fig. 5a). Finally, peptide P26 was singled out as a focus and its binding properties as well as biological activities were examined. In terms of affinity, the results showed that P26 can specifically bind to TIM3 at the cellular and molecular levels. Additionally, P26 can block the TIM3/Gal-9 interaction and compete with Gal-9 to bind TIM3. The competition and blocking effects of P26 and Gal-9 may be due to the shared binding site of P26 and Gal-9 on TIM3, and thus, the occupation by P26 to TIM3 will significantly thwart the recruitment and binding of Gal-9. On the other hand, the topologically structural alteration of TIM3 protein upon the interaction with P26 may lead to the augmented difficulty of TIM3 binding to Gal-9 resulted from the covered or disappeared binding sites. Collectively, the mechanism related to the blockade of TIM3 and Gal-9 by P26 is worthy of exploration. With regard to biological functions and activities, P26 is able to activate CD4 + T cells; notably, P26 also reactivates Gal-9-mediated T-cell suppression in a mock physiological environment (Fig. 5b).

From the in vivo experiment, our results indicate that P26 can inhibit MC38-hPD-L1 tumor growth in mice. The dose of P26 selected for in vivo experiments is 5 mg/kg, which is partly based on the in vitro SPR analysis. Besides, the previous studies offered us some valuable reference on the dosage, which utilized 5 mg/kg [16] or 2–4 mg/kg [17] of peptides.

It should be emphasized that our present study failed to outline P26′s in vivo distribution in tumor and other tissues. We realized that measuring the in vivo levels of P26 and establishing its correlation with the endpoint of tumor growth inhibition are still problematic, which need to be solved in future work of positioning P26 as a therapeutic drug. TIM3 predominates on the surface of T cell existing in the blood and peripheral tissues apart from infiltrated tumor tissues [16–18], which forms a severely big hurdle and abrogates the precise tracing of P26 even with the fluorescence labels. Furthermore, the traditional fluorescent labels are susceptible to multiple factors such as pH and prone to cancelation in the long term [19, 20]. In general, fluorescent dyes with varied colors last amid the scope of delectability within minutes to 7 days under the fixation status [21–23]. For the present study of 36-day observation course, the fluorescence conjugated to the P26 would definitely disappear or formidably fatigue upon excitation. Besides, peptide might be degraded at some sites with the fluorescent probes alive, so the fluorescence from either chemicals or proteins may fail to reflect the authentic status of peptides in vivo [24–26]. Thus, fluorescence labeling of P26 bears the incompatibility in our study. Additionally, mass spectrometry analysis of in vivo levels of P26 may be a viable option, but the procedures require some optimizations. Finally, P26-specific antibody is in the process of development for the purpose of establishing antibody-based detection techniques and assisting the further therapeutic development of P26.

TIM3 has emerged as a promising target for tumor immunotherapy, and a few antibodies against TIM3 have been reported to exhibit excellent effects in enhancing antiviral immune responses, stimulating IFN-mediated anti-tumor immunity of T cells, and counteracting tumor growth [27]. Also, there are some reports on TIM3 inhibitors, but mechanisms of the action are still unclear. It has been shown that the anti-tumor mechanism of anti-TIM3 requires CD4^+^ T cells and CD8^+^ T cells producing IFN-γ, and the model dependence on host CD11^+^ DC is relatively minimal [28]. Plus, it has also been reported that anti-PD-1 immunotherapy could develop resistance due to selective activation of TIM3, leading to immune escape [29]. Additional studies have shown that the use of combined antibodies against TIM3 and anti-PD-1 can prevent resistance to PD-1 therapy and reverse T cell failure and enhance anti-tumor activity of NK cells [17, 30]. In view of these resistance mechanisms, the combination of PD-1 and TIM3 inhibitors after anti-PD-1 treatment may be an effective treatment strategy for reversing anti-PD-1 resistance in the future.

Peptides have the advantages of small molecular mass, low cost, and easy access to synthesis, and are gradually becoming attractive for drug research and development [31]. Studies of related peptides on CTLA-4, PD-1, and PD-L1 have demonstrated the ideal functional activities in tumor immunotherapy [31–34]. However, so far, there have been no reports on the application of peptide inhibitors of TIM3, and the mechanism and development of TIM3-targeting peptides still require further research and optimization. Encouragingly, this study proposes the novel peptide P26 which is involved in the interference of TIM3/Gal-9 interaction and activation of CD4^+^ T cells through increased expression of cytokines (IFN-γ, IL-2). Moreover, in the in vivo experiments, P26 also played a beneficial role in inhibiting tumor growth in MC38-hPD-L1 colon cancer cell-bearing mice. To maximize and optimize the suppressive effect of P26 on tumor, key residues in the P26 peptide sequence and underlying mechanism are worthy of further research. In addition, it is necessary to study the selectivity and specificity of P26, which will assist clinical selection of drugs. Hence, the prospect of peptide P26 is bound to be attractive, and may have the potential to act as an alternative, adjuvant, or supplement to antibody-based therapy in immunotherapy of cancer.

## Data Availability

All data generated or analyzed during this study are included in this published article.
